# Significance of genetic variations in developmental enamel defects of primary dentition in Polish children

**DOI:** 10.1007/s00784-017-2115-1

**Published:** 2017-04-05

**Authors:** Karolina Gerreth, Katarzyna Zaorska, Maciej Zabel, Michal Nowicki, Maria Borysewicz-Lewicka

**Affiliations:** 1 Department of Pediatric Dentistry, Poznan University of Medical Sciences, 70 Bukowska Street, 60-812 Poznan, Poland; 2 Department of Histology and Embryology, Poznan University of Medical Sciences, 6 Swiecickiego Street, 61-781 Poznan, Poland

**Keywords:** Enamel, Developmental defects, Primary teeth, Nursery school, Children

## Abstract

**Objectives:**

The aim of the study was to reveal the association between developmental defects of enamel (DDE) and single nucleotide polymorphisms (SNPs) in the *ENAM, AMELX, AMBN, TUFT1*, and *TFIP11* genes.

**Material and methods:**

The molecular analysis was carried out in 52 children, aged 10–42 months, from four nursery schools situated in the region of Poznan, Poland (26 individuals with hypomineralization and/or hypoplasia of enamel - “cases” and 26 unaffected children - “controls”), chosen from 262 individuals that had prior dental examination. Six selected SNP variants (rs17878486 in *AMELX*, rs4694075 in *AMBN*, rs3796704 in *ENAM*, rs134136 and rs5997096 in *TFIP11*, and rs3790506 in *TUFT1*) were genotyped by the TaqMan probes assay. Genotype and allele frequencies were calculated, and a standard chi-squared analysis was used to test for deviation from Hardy-Weinberg equilibrium. The association between genetic variations and developmental defects of enamel was assessed by the Fisher’s exact test and *p* ≤ 0.05 was considered statistically significant.

**Results:**

Statistically significant positive correlations were found between the rare T allele (*p* = 0.005) and the TT genotype (*p* = 0.0052) for rs17878486 in *AMELX* and occurrence of developmental enamel defects in primary dentition of children. For rs4694075 in *AMBN*, a higher incidence of the rare T allele (*p* = 0.0157) was observed in controls compared to DDE cases, whereas the wild-type CC homozygote was more frequent in DDE cases than in controls (*p* = 0.0062).

**Conclusions:**

The study showed that the single nucleotide polymorphisms in the *AMELX* and *AMBN* genes may be genetic variants that contribute to developmental defects of enamel in primary dentition of children.

**Clinical relevance:**

The single nucleotide polymorphisms of enamel formation genes may increase the risk for developmental defects of enamel (DDE) occurrence in primary dentition in children.

## Introduction

Developmental defects of enamel (DDE) are disturbances in tooth hard tissue matrices and mineralization that occur during odontogenesis and may affect both primary as well as permanent dentition [1]. The etiological factors causing such changes may be localized or systemic and can act in the prenatal, perinatal, or postnatal periods [1].

Developmental defects of enamel are categorized into two main groups, such as enamel hypomineralization and enamel hypoplasia [2, 3]. Hypomineralization (also termed opacity) is a qualitative defect of the enamel appearing as a defect in the translucency of this tissue. The lesions may manifest as demarcated as well as diffuse opacities [2]. Alternatively, hypoplasia is a quantitative defect in the enamel surface related to reduced its thickness [2].

Different data concerning the etiology of developmental enamel defects in primary dentition is reported in the literature [1, 4–7]. The risk of the occurrence of such lesions in deciduous dentition could be affected by numerous factors, such as nutritional problems, social variables, episodes of infections in childhood, low birthweight, prematurity, and problems during pregnancy as well as breastfeeding [4].

However, developmental defects of tooth enamel may not only be caused by environmental factors but also by genetic disturbances [8].

Amelogenesis as well as odontogenesis are under strict genetic control: the shade, shape, size, enamel microhardness, and caries susceptibility can all be affected by genetic variation [9, 10]. Currently, there is scarce information in the literature concerning the influence of genetic variations in genes encoding enamel matrix proteins on the occurrence of developmental enamel defects in primary dentition. Available data mainly shows associations between different genetic variations in genes encoding dental enamel proteins and various forms of amelogenesis imperfecta or Molar-Incisor Hypomineralization (MIH) [10–13]. Moreover, it is possible that genetic variations in genes encoding enamel proteins may also interact in some way with environmental factors. Therefore, much more research on this topic is necessary.

Single nucleotide polymorphisms are useful genetic markers as they are the most frequent type of genetic variation, occurring every 1.3 kb on average in the human genome [14]. Moreover, most SNPs are biallelic, with the alleles differing only at a single nucleotide [14].

Dental enamel is formed by ameloblasts, which are tooth-specific cells that produce and secrete enamel matrix proteins during the secretory stage of amelogenesis [12, 15]. The most significant proteins may be divided into nonamelogenins (enamelin, ameloblastin, and tuftelin) and amelogenin [15]. Subsequently, enamel matrix proteins are exchanged with calcium and phosphate at the maturation stage [15]. During this phase, ameloblasts are primarily responsible for the degradation and reabsorption of enamel matrix proteins. Such events create space for enamel crystals to increase in thickness and width, leading to hardening of dental enamel [12].

Therefore, factors affecting ameloblasts during the secretory stage of amelogenesis can restrict crystal elongation, leading to hypoplastic or pathologically thin enamel [9, 10]. Meanwhile, disturbances throughout the maturation and transitional stages may cause pathologically soft enamel of normal thickness, i.e., hypomineralised or hypomaturated enamel [9].

As amelogenesis is under strict genetic control, it looks reasonable to hypothesize that genetic variation in genes encoding proteins involved in dental enamel formation may contribute to increased risk of developmental enamel defects in primary teeth of children.

## Objectives

The aim of the study was to determine association between genetic variations in genes encoding such enamel proteins as enamelin, amelogenin X, ameloblastin, tuftelin 1, and tuftelin interacting protein 11 (six chosen SNPs within the *ENAM*, *AMELX*, *AMBN*, *TUFT1*, and *TFIP11*) and developmental defects of enamel (DDE) occurrence in a population of nursery school aged Polish children from the region of Poznan (Wielkopolska Province, Central-West Poland).

## Material and methods

### Study group

The survey was carried out between April and June 2014, in the city of Poznan, and involved children who attended four nursery schools from one institution. We chose this institution as it is one of the largest in the city. At the time of the research, 321 children attended the aforementioned nursery schools. Before beginning of the study, consent was obtained from the main head teacher of the institution and from teachers from other divisions. Moreover, parents were provided with a consent form and a pamphlet explaining the purpose of the study. Dental examination and oral swab collection were performed only in those children whose parents gave their informed consent.

The researchers visited each nursery school from 2 to 3 times to carry out check-ups and collect biological material, as some children were absent on the days of examination. Finally, parental consent was obtained for 265 children (82.55%); however, only 262 individuals (81.62%) underwent teeth assessment, as three children were absent at the nursery schools on the day of examination.

In general, individuals in the group with enamel defects (“cases”) and those in the control group (“controls”) were matched by gender. The control group included children with the highest number of erupted teeth to be certain they did not have developmental defects of enamel.

### SNPs selection

Literature data has shown an association between developmental defects of enamel and dental caries, including primary dentition [16–18]. Furthermore, some researches found a correlation between genetic variations within genes encoding enamel matrix proteins and the occurrence of dental caries [19–24]. Therefore, in this study, we chose to assess whether SNPs that were previously identified to be important in the etiology of dental caries in primary dentition were similarly associated with DDE in primary teeth in Polish children [23, 24]. In addition, it must be emphasized that we have not found any information in the literature concerning an association between SNPs of enamel formation genes and developmental defects of enamel (DDE) occurrence in primary dentition in any population of children. Available literature data only shows genetic variations in enamel formation genes in patients with some genetic disorders such as amelogenesis imperfecta. Therefore, it might be the first paper on this topic in the population of healthy children.

### Clinical examination and enamel phenotyping

Prior to dental examination, the examiner underwent calibration, and training exercise, from another experienced dentist concerning the diagnosis of developmental defects of enamel.

The clinical examination was carried out in the classroom. A dental explorer was softly used for assessing the smoothness of the tooth surface. Prior to evaluation of mineralization defects of enamel, gauze was used for drying and cleaning the teeth. Firstly, the tooth surface was assessed in natural light, in children sitting close to the window. Secondly, the changes were estimated in artificial light of a headlamp. Enamel defects were recorded on all surfaces of each tooth available for examination, i.e., interdental spaces were excluded when there was no access for evaluation. Developmental defects of enamel were easily distinguished from white spot lesions on clinical grounds, based on the association of the carious lesion with the location on the tooth and areas of mature plaque. Children with teeth with carious lesions or other changes that made differentiation of enamel defects difficult or impossible were excluded from examination and further analysis. Results were written down on a specially prepared data sheet with the child’s personal data. Developmental defects of enamel were evaluated as hypomineralization and hypoplasia. Lesions were categorized (according to modified DDE index for use in screening surveys) as demarcated opacity, diffuse opacity, hypoplasia, or other defects [25]. The latter category was used when the lesion did not suit any other category and included teeth with both hypoplasia and opacity.

Participation in the study was voluntary. Dental examination was done without any pharmacological preparation and was not performed if the child failed to cooperate or refused to participate.

Of 262 children who underwent dental examination, 29 (11.07%) were diagnosed with developmental defects of enamel.

Finally, the case group comprised 26 children with developmental defects of enamel (14 females—53.85% and 12 males—46.15%), aged from 10 to 36 months (mean age and standard deviation (SD) were 24.92 ± 6.67). The participants had from 6 to 20 erupted teeth (16.23 ± 3.87). The control group included 26 children (13 females—50.00% and 13 males—50.00%) who were completely free of enamel lesions and defects (including dental caries or teeth discoloration), aged from 22 to 42 months (32.50 ± 4.56), with 18–20 erupted teeth (19.69 ± 0.68).

Summary of the developmental defects of enamel in particular teeth of the children included in the study is presented in Table 1.

**Table 1:** Developmental enamel defects in children (using Federation Dentaire Internationale (FDI) Two-Digit tooth-numbering system for primary dentition)

No	Sex	Age (months)	Number of erupted teeth	Developmental defects of enamel (DDE)			
				Demarcated opacities	Diffused opacities	Hypoplasia	Other
1	F	26	16		51,52,61,62		
2	M	10	12	61			51
3	M	23	16	63	83		
4	M	23	14	53			
5	F	31	20			61	
6	F	34	20		61,73,85		
7	F	25	16		54,64,74,84		
8	M	14	15	81			
9	F	31	17		52,51,61,62		
10	M	31	18		52,51,61,62		
11	M	23	16		54,53,52,51,61,62,63,64, 84,83,82,81,71,72,73,74		
12	F	25	20		54,55,64,65		
13	M	26	16		54,53,52,51,61,62,63,64, 84,83,82,81,71,72,73,74		
14	F	34	20	51,61			
15	F	22	16		54,53,52,51,61,62,63,64, 84,83,82,81,71,72,73,74		
16	M	14	8	52,51,61,62, 82,81,71,72			
17	M	20	16	54,52,51,61,62,64, 84,82,81,71,72,74			
18	F	10	6	52,51,61,62			
19	M	15	9			61	
20	M	20	16			51,61	
21	M	28	20	61	55,54,64,65 85,84,74,75		
22	F	29	20	53,63			
23	K	36	19	53			
24	F	31	20	55,54,53,63,64,65			
25	F	27	20	53,63			
26	F	28	16			83	

The selected case and control groups were homogenous with no cultural, ethnic, regional and demographic differences since all individuals lived in the city of Poznan and children other than Caucasian ethnic group were excluded from this study.

Moreover, like the rest of Poland, tap water is not additionally and artificially fluoridated in Poznan, and the state sanitary inspector supervises its quality [26]. In recent years, the fluoride level in drinking water in Poznan has oscillated between 0.1 and 1.0 mg/l: for example, the range was 0.1–0.6 mg/l in 1996, 0.2–1.0 in 1997, and 0.14–0.56 mg/l in the second quarter of 2015 [27, 28].

The level of dioxins and dioxine-like compounds in the environment of Poznan is low. The concentration of pollutants, such as organochlorine pesticides, polychlorinated biphenyls, and polybrominated diphenyl ethers in human samples (human milk, umblical cord serum and maternal serum) from the region of Wielkopolska is one of the lowest in comparison with other European countries [29]. Furthermore, while the Wielkopolska Province is an agricultural region of Poland, prohibition of organochlorine pesticides at the end of 1980s has resulted in their reduced levels in humans [29]. Relatively low levels of polychlorinated biphenyl in human samples are also observed as industrial activity is limited in the area. Finally, the consumption of seafood and marine fish is low in comparison to other regions of the country.

### Samples collection and genomic DNA extraction

Biological material was collected during dental examination from 26 individuals with developmental defects of enamel and from 26 unaffected individuals (i.e., free of such lesions). Samples were obtained from buccal swabs that were provided to each child in sterile packs. The inside of the mouth was rubbed, from each side of both cheeks, at least 10 times. Afterwards, the plastic stick was cut off and the swab was placed inside a 1.5-ml Eppendorf tube. The material was stored at +4 °C in a portable fridge until genomic DNA was extracted (on the same day it was collected) with the EXTRACTME DNA Swab & Semen Kit (Blirt S.A., Gdansk, Poland). Samples were briefly centrifuged at short spin and DNA was purified according to the manufacturer’s instruction. The DNA samples were frozen at −20 °C until further use.

In some cases (i.e., when the quality of the biological material for molecular analysis was poor) the samples were taken up to three times. Samples from three children with enamel defects were excluded, as the material was unsuitable for further analysis despite collecting swabs several times.

### Molecular analyses

We performed genotyping for the following six single nucleotide polymorphisms (SNPs) in five genes encoding dental enamel proteins: rs4694075 in *AMBN*, rs17878486 in *AMELX*, rs134136 and rs5997096 in *TFIP11*, rs3790506 in *TUFT1*, and rs3796704 in *ENAM.* The chromosome location and minor allele frequency details for all six SNP markers are listed in Table 2. Five SNPs (rs4694075, rs17878486, rs134136, rs5997096, and rs3790506) are located in intron regions and one SNP (rs3796704) is located in an exon and, therefore, changes the amino acid sequence of encoded protein. Genotyping for all six SNPs (rs4694075, rs17878486, rs134136, rs5997096, rs3790506, and rs3796704) was performed using the TaqMan SNP Genotyping Assays (Life Technologies) as described earlier [23]. The reactions were carried out in 7900HT Real-Time PCR System (Applied Biosystems). TaqMan Allelic Discrimination data were captured using the SDS software (Applied Biosystems).

**Table 2:** Single nucleotide polymorphisms in genes examined in the study

	Gene	SNP ID	Chromosome position	MAF^a^
1	*AMBN*	rs4694075	4:70601197	0.48
2	*AMELX*	rs17878486	X:11295828	0.08
3	*TUFT1*	rs3790506	1:151565890	0.25
4	*TFIP11*	rs134136	22:26503508	0.35
5	*TFIP11*	rs5997096	22:26499991	0.44
6	*ENAM*	rs3796704	4:70643714	0.14

^a^Minor allele frequency

### Statistical analysis

Genotype and allele frequencies were analyzed using chi-square test. The differences between children with DDE (cases) and healthy subjects (controls) were assessed using Fisher’s exact test. We calculated the odds ratio (OR) according to McHugh [30]. A *p* ≤ 0.05 was considered statistically significant.

## Results

A total of 26 cases (12 males and 14 females) and 26 healthy controls (13 males and 13 females) were included in the study.

Allelic frequencies and genomic distribution of polymorphisms were in the Hardy-Weinberg equilibrium, except for rs4694075 and rs17878486, both in the “cases” group (*p* = 0.0022 and *p* = 0.008969, respectively). Due to low concentration and poor quality of some DNA samples, we did not manage to obtain some genotyping results, specifically for rs4694075 in two cases, for rs3790506 in one case, and for rs5997096 in five cases.

Genotype and allele frequencies, and their association with DDE cases or controls, are shown in Table 3. We observed a positive association between the rare T allele and the CT genotype of rs4694075 with healthy controls. In particular, there was a significantly higher prevalence of the rare T allele and the CT heterozygote, as well as lower prevalence of the wild-type CC homozygote, in the control group. This indicates that the rare T allele of rs4694075 may be protective against DDE in children. For rs17878486, the rare T allele and the TT homozygote were significantly associated with DDE cases. There was also a lower prevalence of the wild-type CC genotype in DDE cases compared to controls. This implies that the rare T allele of rs17878486 may be a strong risk variant for DDE in children. There were no differences in the allele/genotype frequencies based on gender for the two abovementioned SNPs (data not shown). We identified two SNPs that are associated with DDE in primary dentition. While these two SNPs (rs4694075 in *AMBN* and rs17878486 in *AMELX)* are intron variants, they can still influence mRNA transcription and protein folding. Furthermore, these two genes encode the two most abundant proteins found in dental enamel (i.e., ameloblastin and amelogenin).

**Table 3:** Frequencies and odds ratio values for single nucleotide polymorphisms examined in the study

SNP marker	Genotype/Allele	Cases	Controls	OR [95% CI]	*P* value
rs4694075	CC	14 (58.33%)	5 (19.23%)	5.88 [1.6–20.91]	0.0062*
	CT	4 (16.67%)	12 (46.15%)	4.29 [1.14–16.07]	0.0309*
	TT	6 (25.00%)	9 (34.62%)	1.59 [0.47–5.42]	0.4601
	C	32 (66.67%)	22 (42.32%)		
	T	16 (33.33%)	30 (57.69%)	2.73 [1.21–6.16]	0.0157*
rs17878486	CC	7 (26.92%)	15 (57.69%)	3.7 [1.16–11.86]	0.0276*
	CT	6 (23.08%)	8 (30.77%)	1.48 [0.43–5.10]	0.5329
	TT	13 (50.00%)	3 (11.54%)	7.67 [1.84–31.97]	0.0052*
	C	20 (38.46%)	38 (73.08%)		
	T	32 (61.54%)	14 (26.92%)	4.34 [1.9–9.95]	0.005*
rs3790506	AA	3 (12.00%)	4 (15.38%)		
	AG	15 (60.00%)	11 (42.31%)	0.49 [0.16–1.50]	0.2088
	GG	7 (28.00%)	11 (42.31%)	1.89 [0.59–9.07]	0.2877
	A	21 (42.00%)	19 (36.54%)		
	G	29 (58.00%)	33 (63.46%)	1.26 [0.57–2.79]	0.5725
rs134136	CC	7 (26.92%)	13 (50.00%)		
	CT	14 (53.85%)	8 (30.77%)	2.63 [0.84–8.17]	0.0956
	TT	5 (19.23%)	5 (19.23%)	1 [0.25–3.97]	1
	C	28 (53.85%)	34 (65.38%)		
	T	24 (46.15%)	18 (34.62%)	1.62 [0.74–3.57]	0.2318
rs5997096	CC	16 (76.19%)	20 (76.92%)		
	CT	5 (23.81%)	6 (23.08%)	0.96 [0.25–3.73]	0.953
	TT	0 (0.00%)	0 (0.00%)	0.81 [0.02–42.61]	0.9176
	C	37 (88.10%)	46 (88.46%)		
	T	5 (11.90%)	6 (11.54%)	0.97 [0.27–3.41]	0.9562
rs3796704	AA	23 (88.46%)	23 (88,46%)		
	AG	3 (11.54%)	3 (11,54%)	1 [0.19–5.48]	1
	GG	0 (0.00%)	0 (0%)	1 [0.02–52.3]	1
	A	49 (94.23%)	49 (94,23%)		
	G	3 (5.77%)	3 (5,77%)	1 [0.19–5.20]	1

*OR* odds ratio, *CI* confidence interval

*p ≤ 0.05

In addition, we found no significant associations for the other four SNPs (rs134136, rs5997096, rs3790506, and rs3796704) in either the control or case (DDE) groups.

## Discussion

Hart et al. findings suggest that mutations in genes crucial in the process of amelogenesis may be essential for the etiology of local enamel hypoplasia [11]. This implies that genetic factors are much more important in the etiology of localized circumscribed hypoplastic enamel defects than previously believed [11]. Therefore, one may expect a similar situation in cases of enamel hypomineralization.

Different authors have reported that genetic variations in genes responsible for enamel formation may result in amelogenesis imperfecta, which are a heterogenous group of heritable disorders characterized by failure of normal amelogenesis resulting in quantitative and qualitative defects of enamel. Indeed, associations between amelogenesis imperfecta, and genetic defects in the proteins involved in dental enamel formation have been described [11–13]. Poulter et al. revealed that deletion of ameloblastin exon 6 was associated with non-syndromic human amelogenesis imperfecta [13]. Kida et al. found heterogeneous mutations in the enamelin gene were responsible for an autosomal-dominant hypoplastic form of this disorder [31]. Similarly, genetic variations have been associated with Molar-Incisor Hypomineralization (MIH), which is a defect in enamel mineralization that presents as demarcated opacities varying in color from white to yellow/brownish, with sharp demarcation between sound and affected enamel, and is observed in permanent molars and incisors [10]. Jeremias et al. showed that SNPs in genes encoding proteins involved in dental enamel formation may cause MIH in patients [10]. However, there is scarce data concerning genetic variations and developmental defects of enamel in primary dentition [13].

Poulter et al. previously examined five primary teeth (unmatched to genotype) retained by family members with hypoplastic amelogenesis imperfecta after natural exfoliation [13]. SNP genotyping hightlighted a conserved region on chromosome 4 that was shared by all affected individuals and whole sequencing revealed a lack of reads across *AMBN* exon 6 [13]. Interestingly, high-resolution CT scanning revealed that one tooth (tooth 1) showed a normal enamel leyer in terms of mineral density and thickness; whereas mean mineral density in the other four teeth (teeth 2–5) was significantly reduced, and the enamel was pitted, varied in thickness, or absent. Scanning electron microscope analysis revealed that tooth 1 had a prismatic structure characteristic of normal human enamel, while in the other four teeth, thicker areas of enamel either lacked normal prismatic structure or the structures were visible but resembled poorly formed prisms. However, in the current study, no primary teeth of children examined were available for further analysis. Therefore, we could not examine the impact of the six SNPs on the ultrastructure of enamel.

The abovementioned evidence, as well as limited data on the environmental and genetic factors causing developmental anomalies of enamel in primary dentition, encouraged us to examine whether single nucleotide polymorphisms in *ENAM*, *AMELX*, *AMBN*, *TUFT1*, and *TFIP11* genes can affect formation of dental enamel. However, it is difficult to compare our results with those of other authors since we found scarce data concerning SNPs in enamel formation genes and the occurrence of developmental defects of enamel. Moreover, unlike our study, the available data on this topic is limited only to permanent teeth.

In the present study, we found a significant positive correlation between the rare T allele (*p* = 0.005) and the TT genotype (*p* = 0.0052) of rs17878486 in *AMELX* and occurrence of developmental enamel defects of primary dentition in children, and the result was not associated with gender. Interestingly, our previous research indicated the T allele and the TT genotype of rs17878486 in *AMELX* were putative strong risk variants for caries [24]. The prevalence of the rare T allele of rs17878486 was significantly more common in those with caries compared to the control group (OR = 10.2, *p* < 0.0001) [24]. Similarly, there was a higher incidence of the minor TT homozygote of rs17878486 in those with caries compared to the control group (OR = 25, *p* < 0.0001) [24]. However, in the present study, clinical examination revealed that among the 26 children with developmental defects of enamel, dental caries was only diagnosed in four individuals (data not shown).

Patir et al. proved that the wild-type C allele was associated with the occurrence of caries and a more severe form of the disease in Turkish children [Patir et al. 200,820]; however, we found that this same allele was protective against enamel defects in our population. Moreover, rs17878486 in *AMELX* appears to correlate with another SNP (rs3790506 in *TUFT1)*, and both SNPs were previously shown to be associated with *Streptoccocus mutans* infection [19]. However, in our study, the frequency of the rs3790506 in *TUFT1* did not vary between cases and controls (*p* = 0.5725).

Impaired function or decreased amounts of extracellular matrix proteins may result in structural alterations of the enamel such as disorganization of the prisms. As amelogenin constitutes a substantial majority of the extracellular matrix protein in developing tooth enamel and is also suggested to plays a unique role in the control of enamel crystal organization and shape, one could assume that SNPs in *AMELX* could contribute to discrete changes in enamel microstructure [32, 33]. Our results support this hypothesis, as we found particular SNP (rs17878486) seemed to affect tooth enamel formation.

We also found a significant difference in the allele and genotype prevalence of rs4694075 in *AMBN* between DDE cases and controls. We found a higher incidence of the rare T allele (*p* = 0.0157) in controls compared to DDE cases, whereas the wild-type CC homozygote was observed more frequently in DDE cases than in controls (*p* = 0.0062). It is interesting that our previous study showed a stronger association between rs4694075 and rs34538475 of *AMBN* in children with caries (*R* ^2^ = 19) than in controls (*R* ^2^ = 1) [24]. While rs34538475 was significantly associated with caries, no specific haplotype was revealed [24]. In a prior study involving permanent teeth, 163 MIH cases from Turkey (and 82 controls) and 71 MIH cases from Brazil (and 89 controls) were genotyped by the TaqMan method, specifically looking at 11 SNPs [10]. These 11 SNPs occurred in five genes, encoding the following proteins: ameloblastin, enamelin, amelogenin, tuftelin, and tuftelin-interacting protein 11. The authors found associations between MIH and SNPs in *TUFT1*, *ENAM, TFIP11*, and *AMBN* (including rs4694075) in the Turkish population, with regards to both the allele and genotype distributions [10].The other four SNPs tested in our research were not associated with either DDE cases and controls. However, other authors have reported differing results; for example, rs3790506 in *TUFT1* and rs5997096 in *TFIP11* were significantly associated with MIH in Turkish children and rs134136 in *TFIP11* was associated with MIH in Brazilian children [10].

The occurrence of alleles and genotypes for the same SNPs is likely associated with the population, as well as with the susceptibility to a disorder. Moreover, a SNP that demonstrates significant association with a disease occurrence in a study does not have to be the causative variant itself, as it might be in a strong linkage disequilibrium with other true positive SNPs in the genome, not yet revealed. Additional environmental factors may also exist and influence the disorder manifestation.

This study has some limitations. Firstly, there were only 52 children included in total (i.e., in both the cases and control groups). The small size of the study population was limited by the difficulty in confirming which children were affected by DDE. When we had doubts concerning the presence or absence of DDE in a tooth, the child was excluded from further analysis, which led to reduced numbers. In addition, many parents did not agree to dental examination of their children. Moreover, in some cases, even if the samples were taken up to three times, the quality of the biological material for molecular analysis was too poor to carry out assessment. Furthermore, enamel defects could be only diagnosed on all surfaces except interdental surfaces. Therefore, it could be possible that DDE might be not diagnosed in those areas.

Despite these limitations, there is certain strength in the present research, since assessment of developmental defects of enamel was done soon after eruption of teeth. Modric et al. noted that this is the optimal time to assess DDE, as such changes could be later lost due to dental caries, trauma or attrition [34]. Moreover, all children participating in the study attended nursery schools located in one city. Therefore, we could assume that they were living in the same environment. Furthermore, the selected population was homogenous with no regional, demographic, cultural, and ethnic differences, as children of other ethnicy were excluded from the analysis. In addition, all procedures (teeth assessment with oral swab collection and molecular analysis) were performed within a short time frame (three months) and were done under the same conditions.

In the present study, we proved that two SNPs (rs17878486 in *AMELX* and rs4694075 in *AMBN*) in two genes encoding proteins that build up tooth enamel (amelogenin and ameloblastin) are significantly correlated with developmental defects of enamel in Polish children. Although it is difficult to test the actual influence of the allele change on the activity of a protein, it is still valuable to assess these SNPs. By obtaining more precise data concerning SNPs occurrence in a larger homogenous group, we may be able to predict a patient’s genetic susceptibility to DDE in future.

In conclusion, we present for the first time the distribution of polymorphisms in genes encoding proteins of enamel formation in a population of Polish children with developmental enamel defects in primary dentition, as well as in their counterparts without such defects. Among the six polymorphisms studied, two variants (rs4694075 in *AMBN* and rs17878486 in *AMELX*) were found to affect tooth enamel formation.
